# Regulatory Insights From 27 Years of Artificial Intelligence/Machine Learning–Enabled Medical Device Recalls in the United States: Implications for Future Governance

**DOI:** 10.2196/67552

**Published:** 2025-07-11

**Authors:** Wei-Pin Chen, Wei-Guang Teng, C Benson Kuo, Yu-Jui Yen, Jian-Yu Lian, Matthew Sing, Peng-Ting Chen

**Affiliations:** 1 Department of Biomedical Engineering, National Cheng Kung University, No.138, Shengli Rd, North District, Tainan, 701, Taiwan, 886 2757575 ext 63438; 2 Department of Engineering Science, National Cheng Kung University, Tainan, Taiwan; 3 Department of Regulatory and Quality Sciences, University of Southern California, Los Angeles, CA, 90033, United States; 4 Medical Device Innovation Center, National Cheng Kung University, Tainan, Taiwan

**Keywords:** AI/ML-enabled medical device, recall, SaMD, 510(k), FDA, Food and Drug Administration, artificial intelligence/machine learning

## Abstract

**Background:**

Artificial intelligence/machine learning (AI/ML) has revolutionized the health care industry, particularly in the development and use of medical devices. The US Food and Drug Administration (FDA) has authorized over 878 AI/ML–enabled medical devices, reflecting a growing trend in both quantity and application scope. Understanding the distinct challenges these devices present in terms of FDA regulation violations is crucial for effectively avoiding recalls. This is particularly pertinent for proactive measures regarding medical devices.

**Objective:**

This study explores the impact of AI/ML on medical device recalls, focusing on the distinct causes associated with AI/ML–enabled devices compared with other device types. Recall information associated with 510(k)-cleared devices was obtained from openFDA. Three recall cohorts were established: “All 510(k) devices recall,” “software-related devices recall,” and “AI/ML devices recall.”

**Methods:**

Recall information for 510(k)-cleared devices was obtained from openFDA. AI/ML-enabled medical devices were identified based on FDA listings. Three cohorts were established: “All 510(k) devices recall,” “software-related devices recall,” and “AI/ML devices recall.” Root cause analysis was conducted for each recall event.

**Results:**

The results indicate that while the top 5 recall root causes are relatively similar across the 3 control groups, the proportions vary, with AI/ML devices showing a higher impact for 87% of all recalls. Design and development–related factors play a significant role in recalls of AI/ML devices with root causes related to device design and software design accounting for 50% of recalls, emphasizing the importance of thorough planning, user feedback incorporation, and validation during the development process to reduce the probability of recalls. In addition, changes in software, including design changes and control changes, also contribute substantially to recalls in AI/ML devices.

**Conclusions:**

In conclusion, this study provides valuable insights into the unique challenges and considerations associated with AI/ML–enabled medical device recalls, offering guidance for manufacturers to enhance verification plans and mitigate risks in this rapidly evolving technological landscape.

## Introduction

### Background

Artificial intelligence/machine learning (AI/ML) has significantly impacted the health care industry, particularly in the development and use of medical devices. Key contributions include enhancing diagnostic accuracy through the analysis of medical imaging data, enabling predictive analytics for disease outcomes, personalizing treatment plans based on patient characteristics, facilitating remote patient monitoring, expediting drug discovery, and improving surgical procedures through robotics [1,2]. In addition, AI aids in optimizing health care management processes, enhancing cybersecurity for patient data protection, and supporting continuous learning and improvement in medical devices.

**Figure 1.**
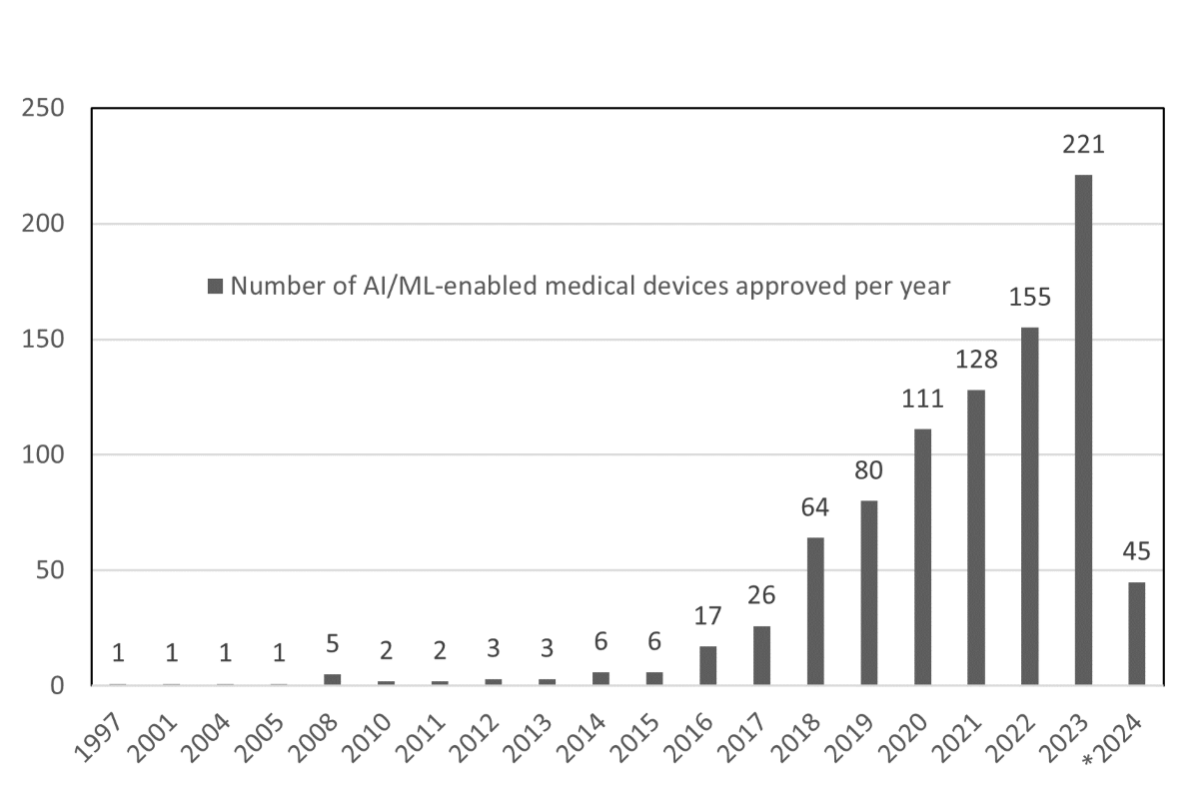
Number of artificial intelligence/machine learning–enabled 510(k) medical devices approved per year from 1997 to March 2024. AI: artificial intelligence; ML: machine learning.

From 1997 to March 2024, the US Food and Drug Administration (FDA) has authorized over 878 Class II AI/ML–enabled medical devices (refer to (Multimedia Appendix 1) ). The quantity may not be large, but from the annual registration records, it can be observed that such products are increasing year by year (refer to Figure 1).

At the same time, the scope of application is also expanding. This trend is reflected in the classification product codes covered by the registered products [2]. Benjamens et al [1] and Zhu et al [2] have successively pointed out in their research that the number of registered AI/ML products has been continuously increasing since 2010. Zhu et al [2] also mentioned that 82% of the registered AI/ML products are concentrated in the two medical specialties of radiology and cardiology, indicating a significant use of digital medical data (such as digital medical imaging data and electrocardiograms) and relevant applications of pattern recognition in diagnosis.

As the FDA approves more and more AI/ML–enabled medical devices, it is critical to understand the unique challenges they pose when it comes to recalls, which are for violations of FDA regulations. This study examines recall data from the FDA, identifying key causes for recalls specifically associated with AI/ML–enabled medical devices. Proactive measures taken for medical devices, Wallace and Kuhn [3] analyzed system failures caused by software faults using recall information and proposed insights for improving software development. Although their analysis was not specifically focused on AI/ML products, FDA data show that one-third of medical devices operated using software are recalled due to software failures. This noteworthy phenomenon has inspired our research thinking.

Recall is a proactive measure aimed at removing or rectifying products that violate laws enforced by the FDA. It is a voluntary action taken by manufacturers and distributors who recognize their responsibility to safeguard public health from products posing risks of injury, significant deception, or other defects [4-7]. Recalls are initiated when a medical device is found to be defective, poses a risk to health, or exhibits both defects and health risks [8]. In rare cases where a manufacturer or importer neglects to voluntarily recall a device jeopardizing health, the FDA may issue a recall order to the manufacturer under 21 Code of Federal Regulations (CFR) 810.

**Figure 2.**
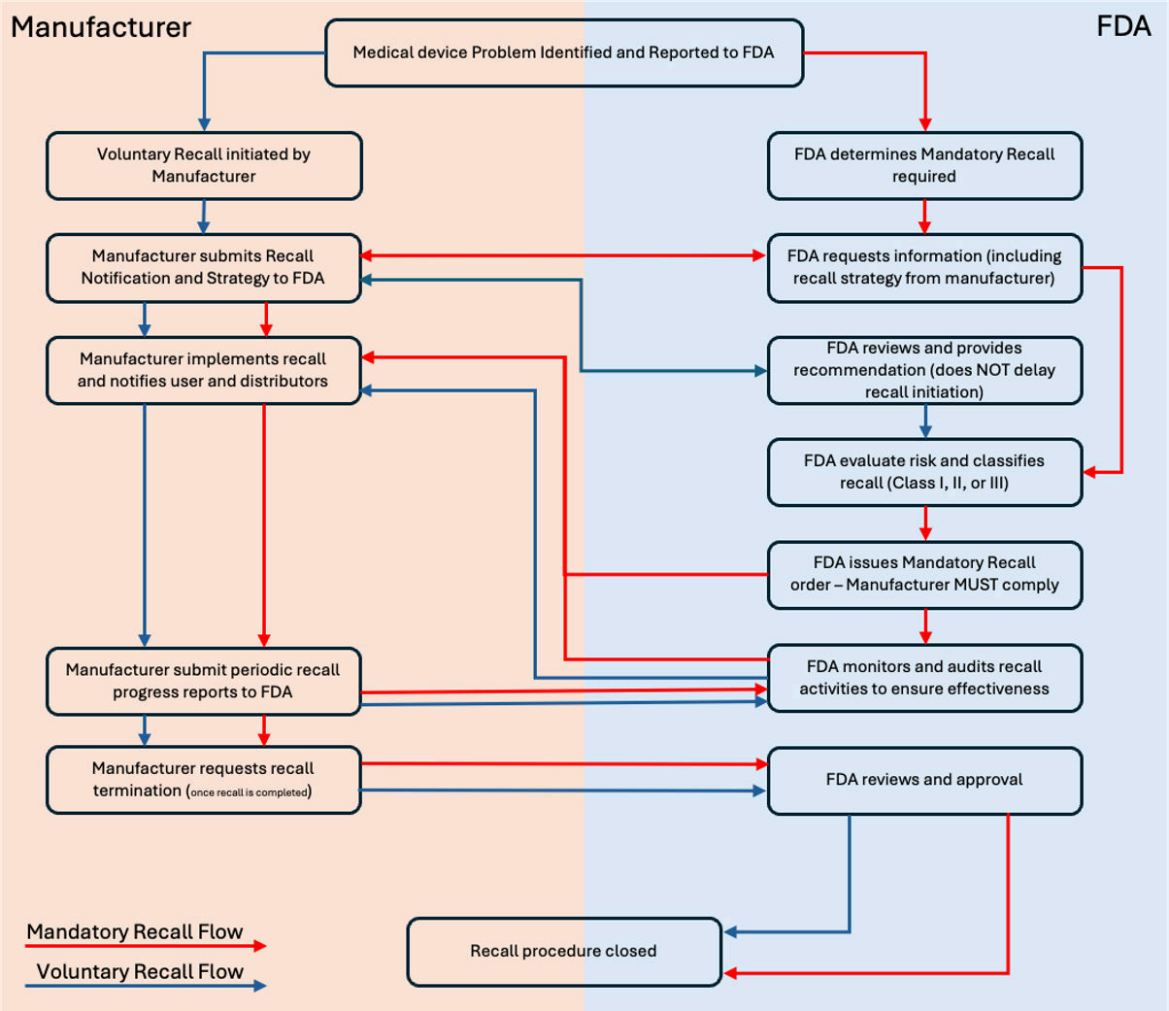
Recall activities with responsible organizations. FDA: Food and Drug Administration.

FDA 21 CFR 7 defines the recall as “a firm’s removal or correction of a marketed product that the FDA considers to be in violation of the laws it administers and against which the agency would initiate legal action, for example, seizure. Recall does not include a market withdrawal or a stock recovery” [9] (refer to Figure 2). Although recalls are intended to ensure patient safety, Zipp [8] points out that the system for initiating recalls often takes a long time to start the recall process [10]. Zipp [8] also uses the example of the recall of Philips sleep apnea ventilator devices to illustrate this situation and proposes using a Unique Device Identifier as a solution to track product flow and expedite the recall process.

### Objectives

This study delves into the recalls linked to AI/ML–enabled medical devices, aiming to discern prevalent recall types unique to these products through comparative analysis with recalls from diverse device types. By scrutinizing recall data, the research endeavors to equip manufacturers with insightful information, enabling them to undertake a more comprehensive evaluation before product introduction. This proactive approach entails implementing measures to preempt root causes associated with recalls, thus mitigating the likelihood of recall incidents. FDA medical device recalls serve as a critical barometer reflecting the intricate landscape of medical device innovation challenges. Each recall unveils potential areas of vulnerability within the innovation process, shedding light on shortcomings in design, development, and regulatory oversight. Recalls highlight the delicate balance between pushing boundaries for technological advancement and ensuring safety and efficacy standards are met. They underscore the complexities of integrating cutting-edge technologies, such as AI/ML, into medical devices while maintaining regulatory compliance. Moreover, these recalls signal the need for continuous vigilance and adaptation in response to evolving risks and emerging technologies, emphasizing the dynamic nature of medical device innovation. As such, FDA recalls provide invaluable lessons for industry stakeholders, guiding them in navigating the intricate terrain of innovation while prioritizing patient safety and regulatory adherence.

## Methods

### Overview

Recall information associated with 510(k)-cleared devices was downloaded from openFDA, a publicly accessible data platform provided by the US FDA to facilitate access to structured regulatory datasets, including medical device recalls [11,12]. AI/ML–enabled medical devices were identified based on the list published by the FDA [13]. These devices were manually screened by the FDA and do not have a specific code to track them. This list comprises medical devices that incorporate AI and ML, based primarily on the information provided within the summary descriptions of their marketing authorizations. The root cause description associated with each recall event was used to characterize the particular recall. Some recall events may be associated with more than one root cause.

In 21 CFR 7.46 (firm-initiated recall), when the initiating firm decides to remove or correct distributed products, reasons for removal or correction must be provided. The Guidance of Initiation of Voluntary Recalls Under 21 CFR Part *7,* Subpart C mentions referencing 21 CFR 820.100(a)(2), which requires procedures for implementing corrections or preventive actions to include an investigation of the causes for nonconformities related to the product, processes, and quality systems. Furthermore, in the FDA regulatory procedures manual, chapter 7 (recall procedures) states that when recalls are classified as Class I or significant Class II recalls, the need for an establishment inspection should be assessed to determine the root cause of the problem and document any potential regulatory actions (7-5-1, 3 Establishment Inspection) [9,14]. Investigation and analysis of the root cause, as outlined in the aforementioned FDA requirements, are a crucial part and one of the important factors for the firm’s decision to remove or correct products. In practice, most companies’ root cause statements align with FDA-recognized categories because they rely on the same industry-accepted terminology. However, the actual analysis and conclusion come primarily from the manufacturer’s quality management system. The FDA’s role is to verify and ensure that the manufacturer’s reasoning makes sense and adequately addresses public health concerns.

Three cohorts were established for comparison and analysis including “All 510(k) devices recall” cohort, “software-related devices recall” cohort and “AI/ML devices recall” cohort (refer to Table 1).

**Table 1.** Characteristics of three recall cohorts.

Root cause description of recall	All 510(k) devices, 167,864 devices			Software-related devices, 3071 devices			AI/ML^a^ devices, 878 devices		
	Recall	Rank	%	Recall	Rank	%	Recall	Rank	%
Component change control	316	22	1	79	18	1	—^b^	—	—
Component design or selection	1472	7	3	305	7	3	1	15	1
Device design	6207	2	14	1405	2	15	13	3	8
Employee error	943	10	2	108	14	1	—	—	—
Environmental control	239	26	1	28	26	0	—	—	—
Equipment maintenance	590	14	1	42	24	0	—		—
Error in labeling	543	15	1	60	19	1		—	—
Finished device change control	26	41	0	5	36	0		—	—
Incorrect or no expiration date	104	35	0	—	—	—	—	—	—
Labeling change control	451	17	1	47	23	0	1	13	1
Labeling design	779	12	2	103	15	1	1	16	1
Labeling false and misleading	304	23	1	18	28	0	—	—	—
Labeling mix-ups	530	16	1	7	34	0	—	—	—
Manufacturing material removal	71	39	0	7	35	0		—	—
Material or component contamination	351	21	1	15	32	0	—	—	—
Mixed-up of materials or components	394	20	1	50	22	1	2	8	1
No marketing application	101	36	0	17	29	0	—	—	—
Nonconforming material or component	4730	3	11	775	4	8	2	11	1
Other	6695	1	16	1115	3	12	2	12	1
Package design or selection	441	18	1	5	38	0	—	—	—
Packaging	866	11	2	17	30	0	—	—	—
Packaging change control	255	25	1	10	33	0	—	—	—
Packaging process control	1031	9	2	19	27	0	—	—	—
Pending	170	29	0	40	25	0	—	—	—
PMA^c^	14	42	0	5	37	0	—	—	—
Process change control	601	13	1	55	20	1	2	9	1
Process control	4717	4	11	507	6	5	11	4	7
Process design	1070	8	2	171	8	2	11	5	7
Radiation Control for Health and Safety Act	207	27	0	140	10	1	1	14	1
Release of material or component before receiving test results	45	40	0	4	39	0	—	—	—
Reprocessing controls	73	38	0	4	41	0	—	—	—
Software change control	126	32	0	126	11	1	3	7	2
Software design	3103	6	7	3103	1	32	68	1	42
Software design manufacturing process	125	33	0	125	12	1	—	—	—
Software design change	157	31	0	157	9	2	4	6	2
Software in the use environment	93	37	0	93	16	1	—	—	—
Software manufacturing or software deployment	111	34	0	111	13	1	—	—	—
Storage	164	30	0	4	40	0	—	—	—
Under investigation by firm	4011	5	9	608	5	6	37	2	23
Unknown or undetermined by firm	411	19	1	51	21	1	—	—	—
Use error	273	24	1	82	17	1	2	10	1
Vendor change control	190	28	0	16	31	0	1	17	1
Total amount	43,100	—	100	9639	—	100	162	—	100

a AI/ML: artificial intelligence/machine learning.

b Zero.

c PMA: premarket approval.

### Data Processing

**Figure 3.**
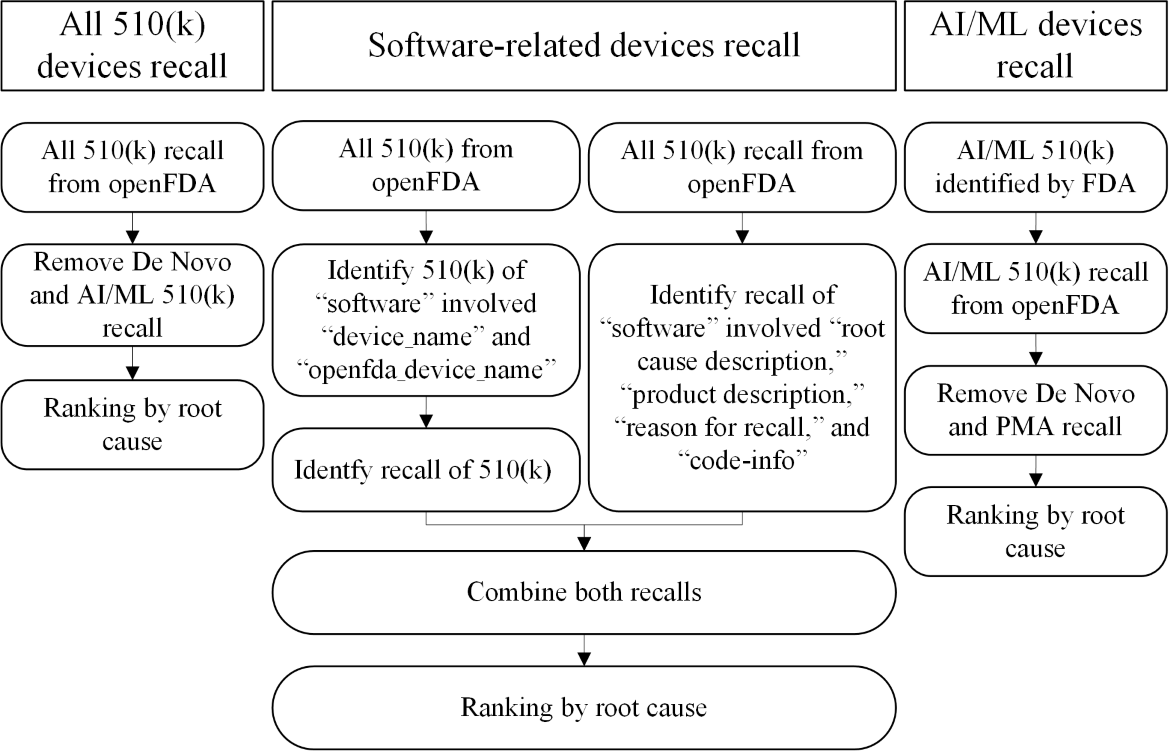
The process for cohort distinction. AI: artificial intelligence; FDA: US Food and Drug Administration; ML: machine learning; PMA: premarket approval.

Each cohort required a specific analytical approach to accurately extract and rank recall events based on their causes. The following numbered list outlines the processes used to collect, filter, and analyze data to derive insights into the prevalence and nature of recalls within each group (refer to Figure 3):

1. All 510(k) devices recall: the “All 510(k) Devices Recall” cohort begins by gathering all 510(k) medical device recall events from the openFDA database [11]. We specifically exclude recalls related to AI/ML devices and De Novo devices to focus on traditional 510(k) recalls. Post exclusion, the recalls are ranked by the frequency of each root cause, helping identify the most common issues within this group.
2. Software-related devices recall: this cohort targets recalls specifically linked to software issues in medical devices. Due to the absence of a direct filter for software-related issues in openFDA, we apply two distinct criteria to accurately capture relevant recalls:
  - Criterion 1: we search within the “device_name” and “openfda_device_name” fields for entries that include the term “Software.” Recalls identified under this criterion were then sequenced by the prevalence of each root cause.
  - Criterion 2: we further refine our search by examining the “root cause description,” “product description,” “reason for recall,” and “code-info” fields for mentions of “Software.” Similar to the first criterion, these recalls were then sequenced by root cause prevalence.
  After identifying and ranking recalls based on both criteria, we merge the results to form a comprehensive sequence of software-related device recalls.

1. AI/ML devices recall: the “AI/ML Devices Recall” cohort consists of recalls involving AI/ML–enabled devices as identified by the FDA [13]. Like the first cohort, we exclude any recalls pertaining to De Novo and premarket approval devices to solely focus on AI/ML 510(k) recalls. These are then ranked by root cause based on occurrence frequency.

### Ethical Considerations

All analyses were conducted using public data available on the FDA medical device databases and from openFDA. Therefore, ethics approval was unnecessary. This study does not include human subject information, primary data collection, or any form of experimentation involving individuals.

## Results

The three cohorts presented in this study are as follows: the “All 510(k) devices recall” cohort includes 167,864 medical devices covering 4473 product codes with a total of 43,100 recalls and 42 root causes. The “software-related devices recall” cohort includes 3071 medical devices, covering 438 product codes with 9639 recalls and 42 root causes. The “AI/ML devices recall” cohort comprises 878 class II medical devices, covering 110 product codes with 162 recalls and 17 root causes (refer to Table 1).

### Root Causes of Recalls

**Figure 4.**
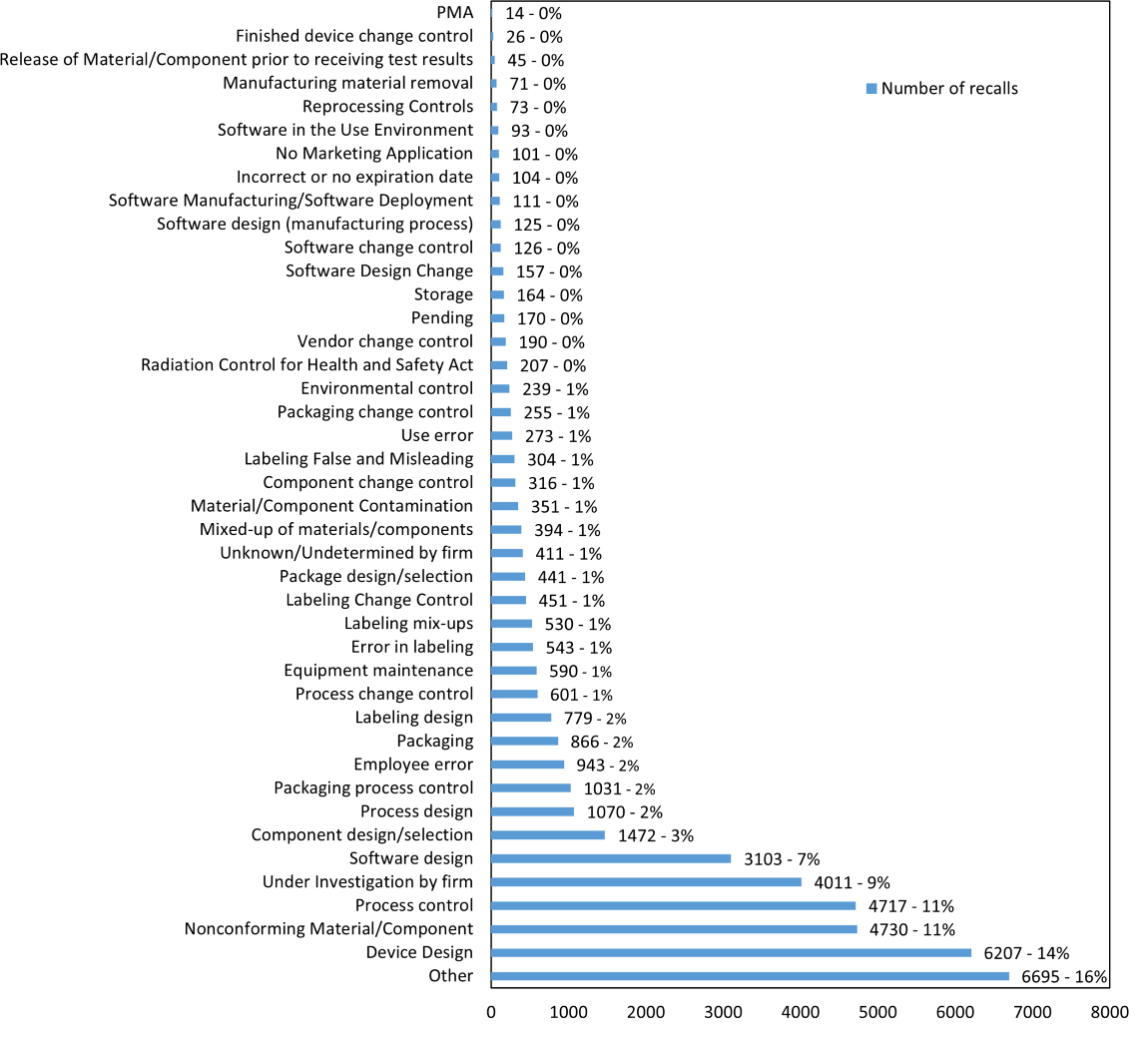
Number of recalls based on the root causes description for “all 510(k) devices”. PMA: premarket approval.

**Figure 5.**
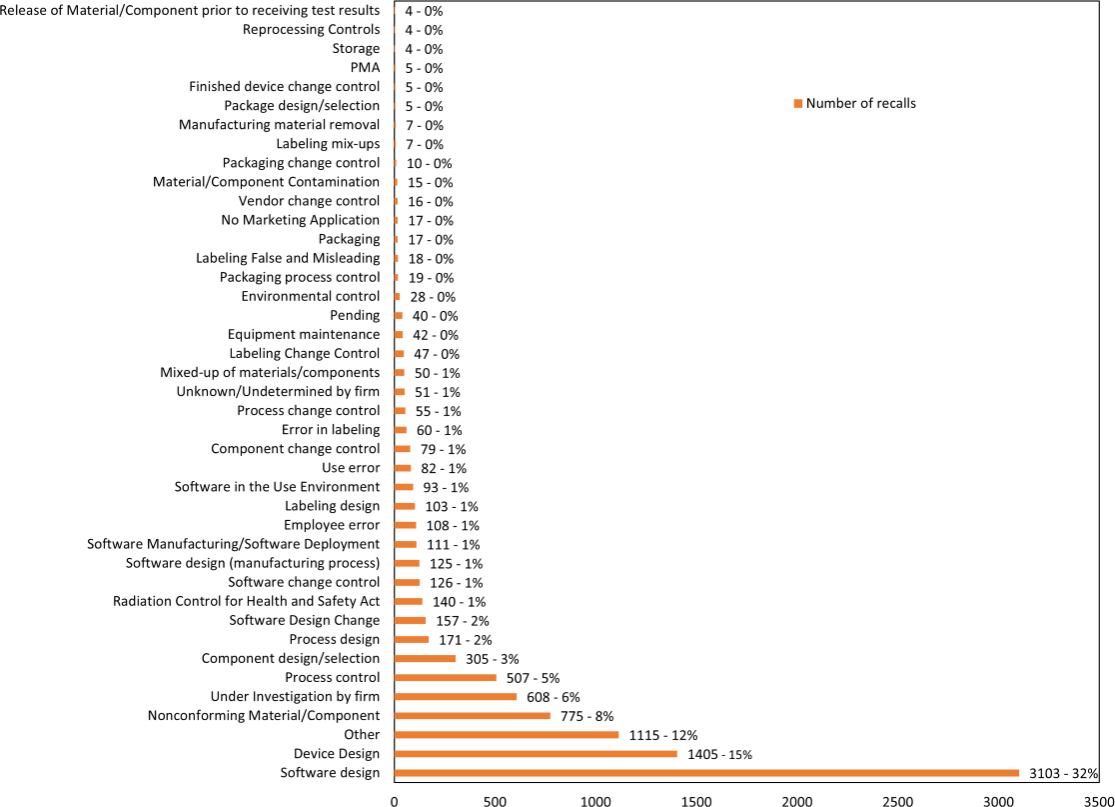
Number of recalls based on the root causes description for “software-related devices.” PMA: premarket approval.

**Figure 6.**
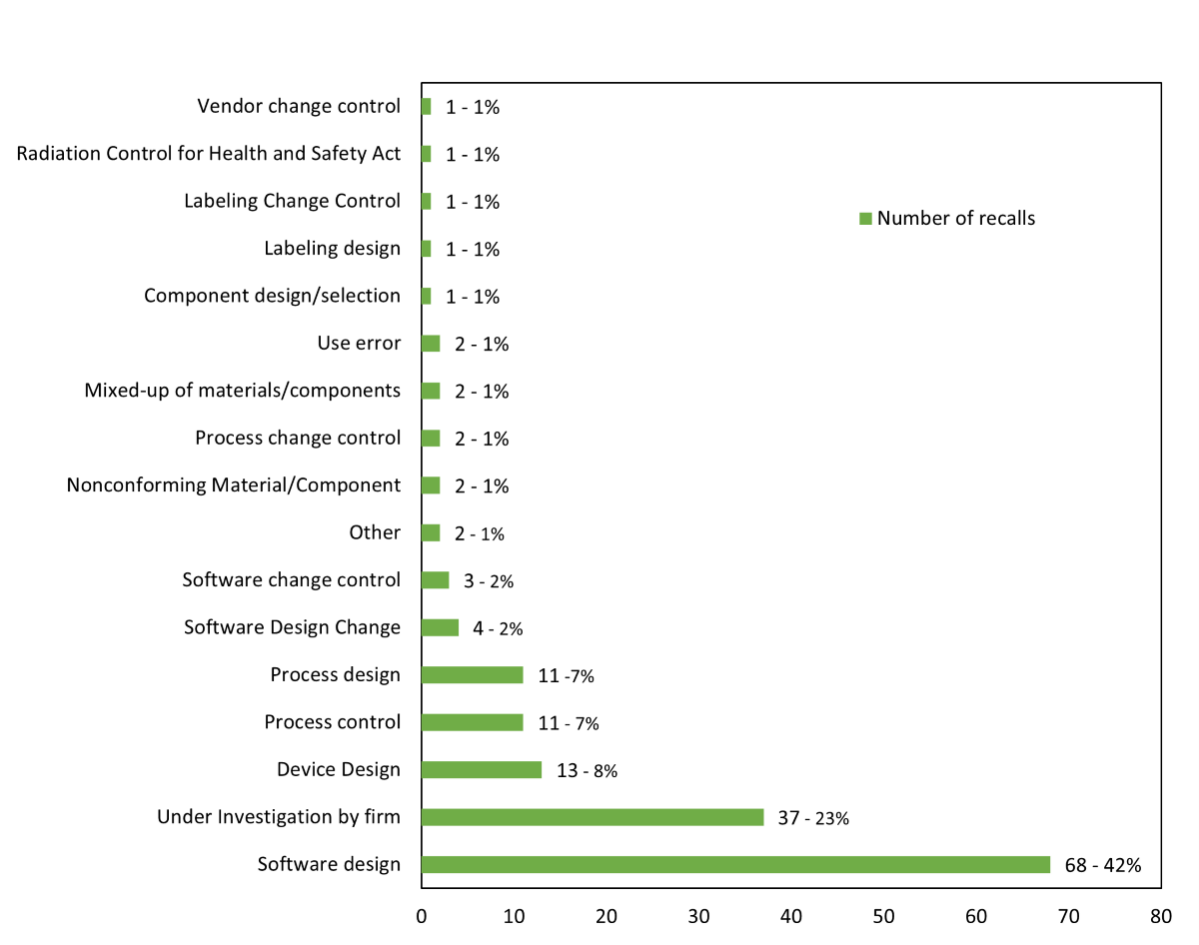
Number of recalls based on the root causes description for “artificial Intelligence/machine learning devices.”

Recalls in both “All 510(k) devices recall” and “software-related devices recall” cohorts that are directly related to software have six common root causes: software design, software design change, software design manufacturing process, software change control, software manufacturing/software deployment, and software in the use environment. The number of recalls for each of these root causes is the same, indicating that the filtering criteria did not miss software-related 510(k) medical devices. In the “AI/ML devices recall” recalls related to software, there are only three root causes: software design, software change control, and software design change. When summing up all software-related root causes, “All 510(k) devices recall” has 3715 recalls, accounting for 9%, “software-related devices recall” has 3,715 recalls, accounting for 39%, and “AI/ML devices recall” has 75 recalls, accounting for 46% (refer to Table 2 and Figures 4-6).

Looking at the number of recalls for each root cause, the top five root causes for “All 510(k) devices recall” are other/6695/16% (recall root cause/number of recall/% of all 510(k) devices recall), device design/6207/14%, nonconforming material-component/4730/11%, process control/4717/11%, and under investigation by firm/4011/9%, totaling 26,360 recalls or 61%. These do not include software-related root causes. For “software-related devices recall,” the top 5 root causes are software design/3103/32%, device design/1405/15%, other/1115/12%, nonconforming material-component/775/8%, and under investigation by firm/608/6%, totaling 7006 recalls or 73%. In “AI/ML devices recall,” the top five root causes are software design/68/42%, under investigation by firm/37/23%, device design/13/8%, process design/11/7%, and process control/11/7%, totaling 140 recalls or 86% (refer to (Multimedia Appendix 2) ).

**Table 2.** Recall events ranked by the number of occurrences for all cohorts.

Root causes description of recall	All 510(k) devices, 167,864 devices			Software-related devices, 3071 devices			AI/ML devices, 878 devices		
	Recall	Rank	%	Recall	Rank	%	Recall	Rank	%
Other	6695^a^	1^a^	16^a^	1115^b^	3^b^	12^b^	2	12	1
Device design	6,207^c^	2^c^	14^c^	1405^c^	2^c^	15^c^	13^b^	3^b^	8^b^
Nonconforming material or component	4730^b^	3^b^	11^b^	775^d^	4^d^	8^d^	2	11	1
Process control	4717^d^	4^d^	11^d^	507	6	5	11^d^	4^d^	7^d^
Under investigation by firm	4011^e^	5^e^	9^e^	608^e^	5^e^	6^e^	37^c^	2^c^	23^c^
Software design	3103	6	7	3103^a^	1^a^	32^a^	68^a^	1^a^	42^a^
Component design or selection	1472	7	3	305	7	3	1	15	1
Process design	1070	8	2	171	8	2	11^e^	5^e^	7^e^
Packaging process control	1031	9	2	19	27	0	—^f^	—	—
Employee error	943	10	2	108	14	1	—	—	—
Packaging	866	11	2	17	30	0	—	—	—
Labeling design	779	12	2	103	15	1	1	16	1
Process change control	601	13	1	55	20	1	2	9	1
Equipment maintenance	590	14	1	42	24	0	—	—	—
Error in labeling	543	15	1	60	19	1	—	—	—
Labeling mix-ups	530	16	1	7	34	0	—	—	—
Labeling change control	451	17	1	47	23	0	1	13	1
Package design or selection	441	18	1	5	38	0	—	—	—
Unknown or undetermined by firm	411	19	1	51	21	1	—	—	—
Mixed-up of materials or components	394	20	1	50	22	1	2	8	1
Material or component contamination	351	21	1	15	32	0	—	—	—
Component change control	316	22	1	79	18	1	—	—	—
Labeling false and misleading	304	23	1	18	28	0	—	—	—
Use error	273	24	1	82	17	1	2	10	1
Packaging change control	255	25	1	10	33	0	—	—	—
Environmental control	239	26	1	28	26	0	—	—	—
Radiation Control for Health and Safety Act	207	27	0	140	10	1	1	14	1
Vendor change control	190	28	0	16	31	0	1	17	1
Pending	170	29	0	40	25	0	—	—	—
Storage	164	30	0	4	40	0	—	—	—
Software design change	157	31	0	157	9	2	4	6	2
Software change control	126	32	0	126	11	1	3	7	2
Software design manufacturing process	125	33	0	125	12	1	—	—	—
Software manufacturing or software deployment	111	34	0	111	13	1	—	—	—
Incorrect or no expiration date	104	35	0	—	—	—	—	—	—
No marketing application	101	36	0	17	29	0	—	—	—
Software in the use environment	93	37	0	93	16	1	—	—	—
Reprocessing controls	73	38	0	4	41	0	—	—	—
Manufacturing material removal	71	39	0	7	35	0	—	—	—
Release of material or component before receiving test results	45	40	0	4	39	0	—	—	—
Finished device change control	26	41	0	5	36	0	—	—	—
PMA^g^	14	42	0	5	37	0	—	—	—
Total amount	43,100	—	100	9639	—	100	162	—	100

a Ranked number 1 root cause of recall for its cohort.

b Ranked number 2 root cause of recall for its cohort.

c Ranked number 3 root cause of recall for its cohort.

d Ranked number 4 root cause of recall for its cohort.

e Ranked number 5 root cause of recall for its cohort.

f Zero.

g PMA: premarket approval.

### Specific Root Causes

If the recalls with software-related root causes are added to the top five root causes, the total recalls for “All 510(k) devices recall” reach 30,075, accounting for 70%, “software-related devices recall” reach 7618, accounting for 79%, and “AI/ML devices recall” reach 147, accounting for 91%. Excluding recalls with software-related root causes, the top five root causes for “All 510(k) devices recall” have 26,360 recalls, accounting for 61%, “software-related devices recall” have 3903 recalls, accounting for 40%, and “AI/ML devices recall” have 72 recalls, accounting for 44% (refer to Table 3 and Multimedia Appendix 3 ).

Furthermore, the first root cause for both “software-related devices recall” and “AI/ML devices recall” is software design with extremely high percentages of 32% and 42%, respectively. In “All 510(k) devices recall,” software design still accounts for 6% of total recalls, ranking sixth. After excluding root causes with unclear definitions, such as other and under investigation by firm, and adding the recall quantities for the second-ranking root cause with significance in the three lists, which is device design, the combined percentage of software design and device design increases to 47% for “software-related devices recall” and 50% for “AI/ML devices recall” reaching half of the total recalls. For “All 510(k) devices recall,” it also reaches 22%, surpassing one-fifth of the total recalls (refer to Table 3).

In terms of product categories, recalls of AI/ML–enabled devices and software-related devices are concentrated in Radiology with another significant category being Cardiovascular. This corresponds to the number of product registrations. Recalls of all 510(k) devices are distributed across a greater number of product categories. However, Radiology and Cardiovascular remain among the categories with higher recall numbers. In addition to these, Orthopedic, General Hospital, and Gastroenterology/Urology are categories with relatively higher recall numbers.

**Table 3.** Statistical comparison of specific root cause recall occurences among different recall cohorts.

Recall with specific root causes	All 510(K) devices		Software-related devices		AI/ML devices	
	Recall	%	Recall	%	Recall	%
Recall amount of all root causes	43,100	100%	9369	100%	162	100%
Recall amount of all software related root cause	3715	9%	3715	39%	75	46%
Recall amount of top 5 recall root causes	26,360	61%	7006	73%	140	86%
Top 5+ all software related root causes	30,075	70%	7618	79%	147	91%
Top 5 - any software related root causes	26,360	61%	3903	40%	72	44%
Device design + software design	9310	22%	4508	47%	91	50%
Top 5 - (other + under investigation by firm)	15,654	36%	5283	55%	103	64%
Recall amount of change related root causes	2122	5%	495	5%	11	7%

## Discussion

### Principal Findings

This study investigated the underlying reasons behind recalls linked to AI/ML–enabled medical devices, pinpointing distinct causes compared with other device types. The first control group encompasses all recalls linked to either a 510(k) device. The second control group encompasses recalls tied to software.

Comparing the root causes and their corresponding recall quantities among the “All 510(k) devices recall,” “software-related devices recall,” and “AI/ML devices recall” groups, it’s evident that the combined recall occurrences for the top five root causes accounted for over 60% across all cohorts [15]. The recall total for “software-related devices recall” even surpasses 73%, while “AI/ML devices recall” reaches 86%. Despite the inclusion of unclearly defined root causes such as “other” and “under investigation by firm” in the top 5, removing these 2 root causes still results in proportions of 36%, 55%, and 64% for “All 510(k) devices recall,” “software-related devices recall,” and “AI/ML devices recall,” respectively. Although the definitions of products in the three control groups differ, it is interesting to note that if the top seven recall root causes are considered, the three groups are not far apart. The ranking of recalls varies slightly, and in the case of “AI/ML devices recall,” process design replaces component design/selection [14]. Improving the top seven recall root causes can have a significant positive impact on recalls and is a topic worthy of further investigation (refer to Table 3).

Looking at recalls related to software, it is clear they constitute a high proportion in “software-related devices recall” and “AI/ML devices recall” accounting for 39% and 46%, respectively. The software design root cause alone accounts for 32% and 42%, respectively with a combined percentage of 82% and 91% for all software-related recall root causes in “software-related devices recall” and “AI/ML devices recall.” Software design can be considered the primary recall root cause for both groups. When adding the device design factor to recalls, the total reaches close to half for both “software-related devices recall” and “AI/ML devices recall,” especially in “AI/ML devices recall,” where it surpasses half, reaching 50%. Including recalls related to process design and labeling design further increases the proportion to 58% [14]. These design and development-related factors leading to recalls likely represent inherent risks present during the product planning or design phase. The impact of these factors on “AI/ML devices recall” is notably higher than in the other 2 control groups. Considering experienced design and development engineers, incorporating user feedback, and implementing thorough validation during the development process could effectively reduce the probability of recalls.

Another group of root causes affecting recalls in “AI/ML devices recall” is related to changes, including software design change, software change control, process change control, vendor change control, and labeling change control. Although the quantities are relatively fewer with 4 root causes, they account for over a quarter (29.4%) of the total root causes, causing 7% of recalls. Compared with the 5% for “All 510(k) devices recall” and 5% for “software-related devices recall,” this is relatively higher. Particularly in “AI/ML devices recall,” where changes in corresponding software are relatively frequent, it is crucial for manufacturers to have comprehensive verification plans after any alterations.

The primary reasons for recalls in AI/ML medical devices include software design errors, nonconforming materials, inadequate process controls, and software implementation issues. Among these, software design errors are the most common, potentially leading to device malfunction, incorrect dosing, and compromised patient safety. Unlike traditional medical devices, AI/ML systems involve dynamic algorithms that evolve through data-driven learning, necessitating specialized validation processes beyond conventional static software testing [1,2]. Moreover, many AI/ML medical device recalls result from implicit assumptions made during algorithm design, such as dosage calculations or diagnostic thresholds. These assumptions must be explicitly identified, documented, and integrated into rigorous validation processes [4]. As a result, AI/ML–enabled medical devices present unique validation and quality assurance challenges, requiring continuous monitoring and adaptive regulatory frameworks to ensure their reliability and safety.

Furthermore, as AI/ML devices adapt postmarket based on new data, continuous market surveillance and real-time feedback loops are imperative to identify risks and respond proactively. Regulatory guidance increasingly emphasizes the necessity of robust monitoring systems, postmarket validation, and rapid recall readiness to address emerging problems [5,9]. These elements should be systematically incorporated into development lifecycle management plans for AI/ML–enabled medical devices.

Specifically, our analysis indicates that the most frequent root causes for AI/ML–enabled device recalls are software design errors, inappropriate software changes, inadequate process controls, and issues in software implementation. These factors can lead to critical patient safety impacts, including incorrect dosing, system malfunction, diagnostic errors, or device stoppage, with the potential for severe adverse health outcomes.

Based on the author’s over 20 years of experience in testing, inspection, and certification of medical devices within the testing, inspection and certification industry, including work with authorized third-party organizations such as US FDA Accredited Person and EU (European Union) Notified Body, the author recommends that manufacturers adopt the following strategies to ensure the long-term safety and reliability of AI/ML–enabled medical devices:

1. Implement specialized validation frameworks, where AI/ML models undergo rigorous adversarial testing, real-world scenario validation, and continual performance monitoring, moving beyond reliance on premarket testing alone,
2. Establish ongoing market surveillance mechanisms, shifting regulatory focus toward total product lifecycle oversight to ensure AI systems remain effective as they evolve with new clinical data, and
3. Integrate user-centric risk mitigation practices, such as proactive feedback loops, to enable rapid adjustments and software updates that address emerging risks in clinical settings.

These strategies will better align regulatory oversight with the evolving nature of AI/ML technologies, ensuring both patient safety and long-term technology sustainability.

### Limitation

This study has several limitations. First, the FDA currently does not have specific product codes or identification methods for Software as a Medical Device or software-related medical devices. Therefore, the data used in this study were derived from existing FDA data related to software to identify and analyze recall data for software-related medical devices as comprehensively as possible. Second, AI/ML–enabled devices also lack specific product codes or identification methods. Although the FDA manually updates its registry of AI/ML–enabled medical devices, this process is irregular, potentially leading to omissions in the data. These limitations restrict the ability to fully capture and analyze the unique challenges of AI/ML–enabled medical devices, such as specialized algorithm validation, handling of implicit assumptions, and algorithm performance variability. In addition, the publicly available FDA recall databases lack comprehensive information on software-specific faults, manufacturer-specific internal verification processes, and the complexity inherent in AI/ML technologies, including algorithm adaptability over time, real-world performance drifts, and user interaction dynamics.

### Future Work

To address these limitations, future research should incorporate direct interactions with device manufacturers, internal company data, qualitative insights from developers, and longitudinal analyses to better explore these challenges and validate effective risk management strategies [10,14,16,17]. Based on the results of this study, we also plan to further investigate each recall of AI/ML–enabled medical devices and the reported reasons behind them to conduct a more in-depth analysis.

We hope to systematically deduce the real factors leading to recalls and compare them with current regulatory requirements to see if there is a need to adjust or strengthen regulatory requirements to reduce the probability of recalls and enhance public safety.

As AI/ML continues to reshape medical practices, a proactive and informed approach to regulatory oversight and device development becomes imperative for ensuring the safety and efficacy of these transformative technologies.

### Conclusion

As the FDA authorizes an increasing number of AI/ML–enabled medical devices, it becomes crucial to understand the distinct challenges they pose in terms of recalls. This study delves into the intricate landscape of medical device recalls, focusing specifically on those associated with AI and ML. Comparisons across three control groups, namely “All 510(k) devices,” “Software-related devices,” and “AI/ML devices,” unveil similarities in the top 7 recall root causes with variations in proportions. Notably, AI/ML devices exhibit a higher impact, especially concerning design and development-related factors. This study underscores the importance of robust planning, user feedback incorporation, and thorough validation during the development process to mitigate the inherent risks associated with AI/ML–enabled medical devices. Recalls related to software, particularly software design, constitute a significant proportion in both “software devices” and “AI/ML devices.” Changes in software also emerge as a notable contributor to recalls in AI/ML devices, underscoring the need for comprehensive verification plans after alterations.

In addition to standard regulatory compliance, AI/ML–enabled medical devices face unique validation challenges due to their dynamic nature and reliance on continuous learning algorithms. Such devices require specialized validation strategies that accommodate algorithm adaptability and evolving real-world performance [4]. Continuous market analysis and proactive user feedback integration are also particularly crucial for these devices, as they help detect and address issues promptly, thereby preventing recalls related to software logic errors, data-driven failures, or algorithmic inaccuracies [3,4].

## Supplementary material

10.2196/67552 Multimedia Appendix 1 Food and Drug Administration artificial intelligence/machine learning–enabled medical devices list.

10.2196/67552 Multimedia Appendix 2 Number of recalls from the top 5 root case for (a) all 510(k) devices recall, (b) software-related devices recall, and (c) artificial intelligence/machine learning devices recall.

10.2196/67552 Multimedia Appendix 3 Number of recall with specific root causes for (a) all 510(k) devices, (b) software-related devices, and (c) artificial intelligence/machine learning devices.

## Abbreviations

AI: artificial intelligence
CFR: Code of Federal Regulations
EU: European Union
FDA: Food and Drug Administration
ML: machine learning
