# Supplementary figures and images for: Regulatory Insights From 27 Years of Artificial Intelligence/Machine Learning–Enabled Medical Device Recalls in the United States: Implications for Future Governance
Source: JMIR Med Inform. 2025 Jul 11;13:e67552. doi: 10.2196/67552 (PMC12274014; doi:10.2196/67552)

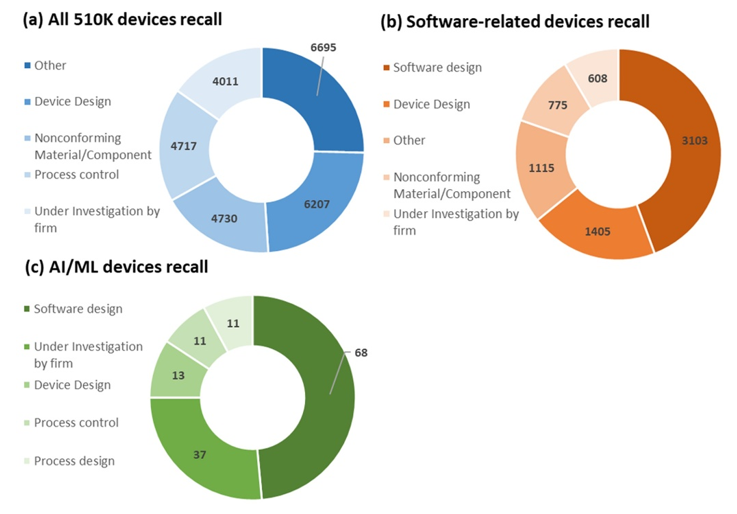

Supplement: Multimedia Appendix 2 [file medinform-v13-e67552-s002.png]

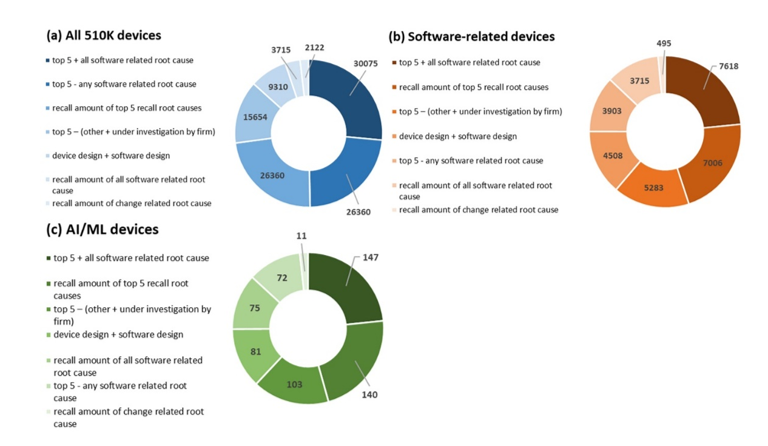

Supplement: Multimedia Appendix 3 [file medinform-v13-e67552-s003.png]
